# Aldo-keto reductase family 1 member C1 regulates the osteogenic differentiation of human ASCs by targeting the progesterone receptor

**DOI:** 10.1186/s13287-021-02425-3

**Published:** 2021-07-07

**Authors:** Xuenan Liu, Xiaomin Lian, Xuejiao Liu, Yangge Du, Yuan Zhu, Menglong Hu, Ping Zhang, Yunsong Liu, Yongsheng Zhou

**Affiliations:** Department of Prosthodontics, Peking University School and Hospital of Stomatology, National Laboratory for Digital and Material Technology of Stomatology, Beijing Key Laboratory of Digital Stomatology, National Clinical Research Center for Oral Diseases, 22 Zhongguancun South Avenue, Beijing, 100081 People’s Republic of China

**Keywords:** AKR1C1, Osteogenesis, Adipose-derived mesenchymal stromal/stem cells, Progesterone receptor

## Abstract

**Background:**

As a promising way to repair bone defect, bone tissue engineering has attracted a lot of attentions from researchers in recent years. Searching for new molecular target to modify the seed cells and enhance their osteogenesis capacity is one of the hot topics in this field. As a member of aldo-keto reductase family, aldo-keto reductase family 1 member C1 (AKR1C1) is reported to associate with various tumors. However, whether AKR1C1 takes part in regulating differentiation of adipose-derived mesenchymal stromal/stem cells (ASCs) and its relationship with progesterone receptor (PGR) remain unclear.

**Methods:**

Lost-and-gain-of-function experiments were performed using knockdown and overexpression of AKR1C1 to identify its role in regulating osteogenic and adipogenic differentiation of hASCs in vitro. Heterotypic bone and adipose tissue formation assay in nude mice were used to conduct the in vivo experiment. Plasmid and siRNA of PGR, as well as western blot, were used to clarify the mechanism AKR1C1 regulating osteogenesis.

**Results:**

Our results demonstrated that AKR1C1 acted as a negative regulator of osteogenesis and a positive regulator of adipogenesis of hASCs via its enzyme activity both in vitro and in vivo. Mechanistically, PGR mediated the regulation of AKR1C1 on osteogenesis.

**Conclusions:**

Collectively, our study suggested that AKR1C1 could serve as a regulator of osteogenic differentiation via targeting PGR and be used as a new molecular target for ASCs modification in bone tissue engineering.

**Supplementary Information:**

The online version contains supplementary material available at 10.1186/s13287-021-02425-3.

## Background

Bone defect caused by a lot of diseases, such as trauma, tumors, congenital malformation, and inflammation, brings physical and psychological hurts to the patients, as well as heavy economic burden to the family and society [1]. Although emergence of bone tissue engineering provides a promising way to repair bone defect, there is still a lot of work to do to promote bone regeneration [2–4]. As a frequently used seed cell in bone tissue engineering, human mesenchymal stromal/stem cells (hMSCs), such as hASCs and hBMMSCs, can differentiate into osteoblasts, adipocytes, and chondrocytes [5, 6]. How to promote the osteogenesis of hMSCs has become a key point in bone tissue engineering [2, 4]. Compared to hBMMSCs, hASCs has more extensive sources and caused less trauma. Thus, searching for new targets to modify hASCs and promote hASCs to differentiate towards osteoblasts has been a hot topic now.

Human aldo-keto reductases are a big family that take an important part in aldehyde and ketone metabolism [7–11]. Among them, aldo-keto reductase family 1 member C1-C4 and D1 play essential roles in the metabolism of steroid hormones like androsterone and progesterone [7, 10, 12]. Previous studies mainly focus on their roles in the field of tumors [12–15] and have demonstrated that AKR1C1 has close relationship with a lot of sex hormone related tumors, such as ovarian cancer and bladder cancer [12, 16]. But whether AKR1C1 and other members of AKR1 family can regulate osteogenic differentiation of hASCs, as well as how AKR1C1 regulates osteogenesis of hASCs, remain unclear.

As the corresponding receptor of progesterone, progesterone receptor (PGR) mediates progesterone intracellular signal transduction and thus participates in a lot of cell biological activities related to progesterone [17–21]. It has been proved that sex hormones and their corresponding receptors such as estrogen receptor and androgen receptor play an important as well as relatively clear role in regulation of bone homeostasis [22, 23], and they are responsible for sexual dimorphism in bone mass acquisition [22, 23]. Yet, the role of PGR in bone is less clear [24–26]. In vivo experiments using PGR condition KO mice demonstrated that PGR’s function differs in different stage of osteoblast [24], different kinds of bone (trabecular bone and cortical bone) [25], and different genders [24]. Different studies have not reached a consensus on the same osteogenic index, such as cortical bone mass. Meanwhile, although progesterone is one of the main substrates catalyzed by AKR1C1 [27, 28], the relationship between PGR and AKR1C1 has been rarely documented.

Based upon these, we aimed to investigate the critical role of AKR1C1 in osteogenic differentiation of hASCs and its potential mechanism in the present study.

## Methods

### Culture, osteogenic induction, and adipogenic induction of hASCs

Primary hASCs were obtained from ScienCell Company (San Diego, CA, USA). All cell-based in vitro studies were repeated three times using hASCs from three healthy donors. DMEM, FBS, and 100× penicillin and streptomycin mixture were obtained from Gibco (Grand Island, NY, USA). Human ASCs were cultured with 5% CO_2_ atmosphere at 37 °C in proliferation medium (PM), which consisted DMEM, 10% (v/v) FBS and penicillin/streptomycin. The OM comprised DMEM containing 10% (v/v) FBS, penicillin/streptomycin, 10 nM dexamethasone, 10 mM β-glycerophosphate, and 0.2 mM L-ascorbic acid. The AM was comprised of DMEM containing 10% (v/v) FBS, penicillin/streptomycin, 10 μM insulin, 100 nM dexamethasone, 200 μM indomecin, and 500 μM 3-isobutyl-1-methylxanthine (IBMX).

### Lentiviral transfection

Lentiviruses targeting *AKR1C1* (sh*AKR1C1*-1 and sh*AKR1C1*-2) and negative control (NC) were obtained from GenePhama Co. (Suzhou, China). The sequences were as follows: sh*AKR1C1*-1, AGCTTTAGAGGCCACCAAAT, and sh*AKR1C1*-2, ATGTTGACCTCTACCTTATTC. First, hASCs were transfected with lentivirus packaged plasmids in the presence of 5 μg/mL polybrene. After that, transfected cells were screened by puromycin to establish stable knockdown cells.

### RNA interference and plasmid transfection

The sequences of short interfering (si) RNAs targeting PGR (siPGR-1, siPGR-2) and the negative control (siNC) were as follows: siPGR-1, 5′-GCUGCUGGAAGACGAAAGUUA-3′ (sense), 5′-UAACUUUCGUCUUCCAGCAGC-3′ (antisense), siPGR-2, 5′-GCUGCACAAUUACCCAAGAUA-3′ (sense), 5′ UAUCUUGGGUAAUUGUGCAGC-3′ (antisense) and siNC: 5′-UUCUCCGAACGUGUCACGUTT-3′ (sense), and 5′-ACGUGACACGUUCGGAGAATT-3′ (antisense) were purchased from Sangon Co (Shanghai, China). Cells expressing PGR were generated by transfection of pcDNA3-hPR-A and pcDNA3-PR-B into AKR1C1 knockdown cells (sh*AKR1C1*-1 and sh*AKR1C1*-2). Lipofectamine 3000 (Invitrogen, Carlsbad, USA) was used as a transfection agent according to the manufacturer instructions. Cells were collected and gene expressions were analyzed 48 h after transfection.

### Cell proliferation assay

Cells were seeded in 12-well plates at the density of 2 × 10^4^ cells per well. Three wells of the same kind of cells were tested daily from day 1 to day 7. A Cell Counting Kit-8 (CCK8, Dojindo Laboratories, Kumamoto, Japan) was used to count the cells, and growth curves were obtained according to the cell number.

### Alkaline phosphatase staining

After 7 days of culture in PM or OM, the cells were first washed with PBS, and then fixed in ethanol and washed with PBS again. An alkaline phosphatase (ALP) Staining Kit (CWBIO, Beijing, China) was then used to stain the cells. Lastly, the images were scanned.

### Quantification of ALP activity

ALP activity quantification was performed as previously described [29]. Briefly, after 7 days of culture in PM or OM, the cells were washed with PBS and then lysed with 1% TritonX-100 on ice. The total protein concentration was measured by a BCA protein assay kit (Pierce Thermo Scientific, Waltham, MA, USA). The ALP activity was measured by an ALP assay kit (Nanjing Jiancheng Bioengineering Institute, Nanjing, China) and normalized to the total protein content of each sample.

### Alizarin red S staining and quantification

After 21 days of culture in PM or OM, cells were washed with PBS, fixed with ethanol, and then washed with distilled water. After that, 2% Alizarin red S (ARS) was used for staining. Lastly, the images were obtained by a scanner.

For ARS quantification, 100 mM cetylpyridinium chloride was used to solubilize the stained cells. The OD value of each well was then measured spectrophotometrically at 562 nm.

### Oil red O staining

Oil red O staining was performed as previously described [30]. Briefly, after 21 days of culture in PM or AM, the cells were fixed with 10% formalin, rinsed with 60% isopropanol, and stained with 0.3% oil red O working solution. Next, the cells were washed in distilled water, and photographed under a microscope.

### Quantitative real-time reverse transcription PCR

Total RNA extraction, purity and concentration determination, and reverse transcription were performed as previously described [29]. SYBR Green Master Mix (Roche Applied Science, Mannheim, Germany) and a 7500 Real-Time PCR Detection System (Applied Biosystems, Foster City, CA, USA) were used to test mRNA expression by qPCR. Glyceraldehyde-3-phosphate dehydrogenase (GAPDH) was chosen as the reference gene. The primer sequences of human *GAPDH*, *AKR1C1*, *PGR*, *AR*, *ALP*, *RUNX2*, *BGLAP*, *PPARγ*, and *CEBPα* used for qRT-PCR were as follows: *GAPDH* (forward) 5′-GAAGGTGAAGGTCGGAGTC-3′ and (reverse) 5′-GAAGATGGTGATGGGATTTC-3′; *AKR1C1*, (forward) 5′-ATTTGCCAGCCAGGCTAGTG-3′ and (reverse) 5′-AGAATCAATATGGCGGAAGCC-3′; *PGR*, (forward) 5′-GCATCAGGCTGTCATTATGG-3′ and (reverse) 5′-AGTAGTTGTGCTGCCCTTCC-3′; *AR*, (forward) 5′- AATTGTCCATCTTGTCGTCTTCGG-3′ and (reverse) 5′-GCCTCTCCTTCCTCCTGTAGTTTC-3′; *RUNX2*, (forward) 5′-CCGCCTCAGTGATTTAGGGC-3′ and (reverse) 5′-GGGTCTGTAATCTGACTCTGTCC-3′; *BGLAP*, (forward) 5′-CACTCCTCGCCCTATTGGC-3′ and (reverse) 5′-CCCTCCTGCTTGGACACAAAG-3′; *PPARγ*, (forward) 5′-GAGGAGCCTAAGGTAAGGAG-3′ and (reverse) 5′-GTCATTTCGTTAAAGGCTGA-3′; and *CEBPα*, (forward) 5′-GGGCCAGGTCACATTTGTAAA-3′ and (reverse) 5′-AGTAAGTCACCCCCTTAGGGTAAGA-3′.

### Western blot

Western blot was performed as previously described [29]. Briefly, the cells were lysed in radioimmunoprecipitation assay (RIPA) buffer consisting 2% protease inhibitor cocktail (Roche) and 1% phosphatase inhibitor (Roche) to obtain the protein. After that, BCA protein assay kit was used to test the protein concentrations. Thirty-five micrograms of total protein of each sample was subjected to SDS-PAGE. After electrophoresis, proteins were transferred to a polyvinylidene fluoride membrane (Millipore, Billerica, MA, USA). The membrane was blocked with 5% nonfat milk and incubated with anti-AKR1C1 (Abcam, Cambridge, UK), anti-PPARγ, anti-RUNX2, anti-PGR, anti-AR, or anti-GAPDH (Cell Signaling Technology, Beverly, MA, USA) in Tris-buffered saline-Tween 20 (TBST) at 4 °C overnight. After that, the membrane was washed with TBST buffer, incubated with goat anti-rabbit IgG or goat anti-rat IgG (Abcam), and then washed with TBST again. At last, an ECL Western blot kit (CWBIO) was used to visualize the bands.

### Ovariectomy and sham operations

All in vivo experiments in this study were approved by the Institutional Animal Care and Use Committee of the Peking University Health Science Center (LA2019019), and all experiments were performed under the approved guidelines.

Female C57BL6 mice (8-weeks-old (*n* = 12)) were firstly randomly divided into two groups. Pentobarbital sodium (50 mg·kg^−1^) was used for general anesthesia followed by a bilateral ovariectomy (OVX) or sham operation according to standard methods [31].

### Micro-computed tomography and bone morphometric analysis of mice

The femurs of the mice were collected and fixed in 10% formalin for 24 h before washed with 10% sucrose solution. After that, micro-CT images and three-dimensional (3D) reconstructions were obtained as previously described [29]. An Inveon Research Workplace (Siemens) was used to measure and calculate these following parameters: bone volume/total volume (BV/TV), trabecular number (Tb.N), trabecular thickness (Tb.Th), and trabecular separation (Tb.Sp) in the region of interest (0.5 to 1 mm distal to the proximal epiphysis) [32].

### Heterotypic bone and adipose tissue formation assay in vivo

For heterotypic bone formation, the hASCs stably infected with NC, sh*AKR1C1*-1, sh*AKR1C1*-2, or AKR1C1 knockdown cells transfected with vector, wild type plasmid, or mutant plasmid were incubated with tricalcium phosphate (TCP) carrier (Bicon, Boston, MA, USA) scaffolds at 37 °C for 1 h, followed by centrifugation at 150×*g* for 5 min. The hASCs-scaffolds hybrids were implanted into the dorsal subcutaneous space of the nude mice (6-week-old, 6 mice per group). The samples were harvested 8 weeks after implantation and analyzed by H&E and Masson staining.

Heterotypic adipose tissue formation was performed as previously described [30]. Briefly, the cells were cultured in AM for 7 days before implantation, and Collagen Sponge were used as scaffolds instead of TCP. The implants were carefully harvested after six weeks. Next, each sample was cut in half for H&E staining and oil red O staining.

Histologic analysis was performed by examiners blinded to the treatment group.

### Statistical analysis

SPSS Statistics 20.0 software (IBM) was used to perform the statistical analyses. Independent two-tailed Student’s t tests, one-way ANOVA, and Tukey’s post hoc test were used to analyze the comparisons. Data was shown in the form: mean ± standard deviation (SD) of 3 to 10 experiments per group. Values of *p* < 0.05 were considered statistically significant.

## Results

### AKR1C1 was related with the lineage commitment of MSCs

**Fig. 1:**
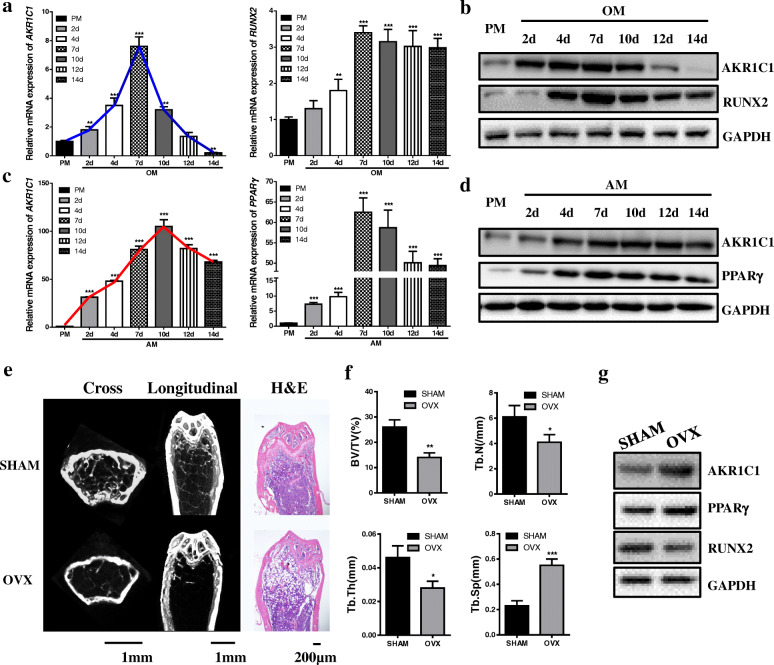
AKR1C1 was a potential target for lineage commitment regulation of MSCs. **a**, **b** The expression of AKR1C1 on different days during the osteogenic induction process tested by qRT-PCR (**a**) and western blot (**b**). **c**, **d** The expression of AKR1C1 on different days during the adipogenic induction process tested by qRT-PCR (**c**) and western blot (**b**). **e** Micro-CT and H&E staining images of femurs in SHAM and OVX mice. **f** Bone morphology analysis of femurs in SHAM and OVX mice based on micro-CT. **g** Western blot of protein expression of AKR1C1, PPARγ, and RUNX2 in BMMSCs obtained from SHAM and OVX mice. *p < 0.05, **p < 0.01, ***p < 0.001

To study the relationship between AKR1C1 and lineage commitment of MSCs, we first tested both the mRNA and protein expression of AKR1C1 on different days during osteogenic induction process. The result indicated that the mRNA expression of AKR1C1 initially increased and subsequently decreased during the osteogenesis of hASCs (Fig. 1a). On the 14th day of osteogenesis, the expression of AKR1C1 was significantly down regulated compared with PM group. Western blot displayed the similar result to that of qRT-PCR (Fig. 1b). Meanwhile, adipogenic induction also led to expression changes of AKR1C1. During the adipogenesis of hASCs, the expression of AKR1C1 was continuously upregulated, determined both by qRT-PCR (Fig. 1c) and western blot (Fig. 1d). Since bone marrow is the only tissue where bone and fat coexist in the same microenvironment, it is an excellent window to study stem cell lineage commitment. So, to further study if AKR1C1 was related to the fate determination of MSCs in vivo, we compared the status of AKR1C1 in the bone marrow mesenchymal stromal/stem cells (BMMSCs) of OVX mice and SHAM mice. Micro-CT and H&E staining showed trabecular bone loss in the femurs of OVX mice (Fig. 1e), which was also confirmed by bone morphology analysis (Fig. 1f) based on Micro-CT. Western blot indicated that compared with BMMSCs of SHAM mice, BMMSCs of OVX mice exhibited elevated expression of AKR1C1 (Fig. 1g).

### AKR1C1 regulated osteogenic and adipogenic differentiation of hASCs in vitro via its enzyme activity

**Fig. 2:**
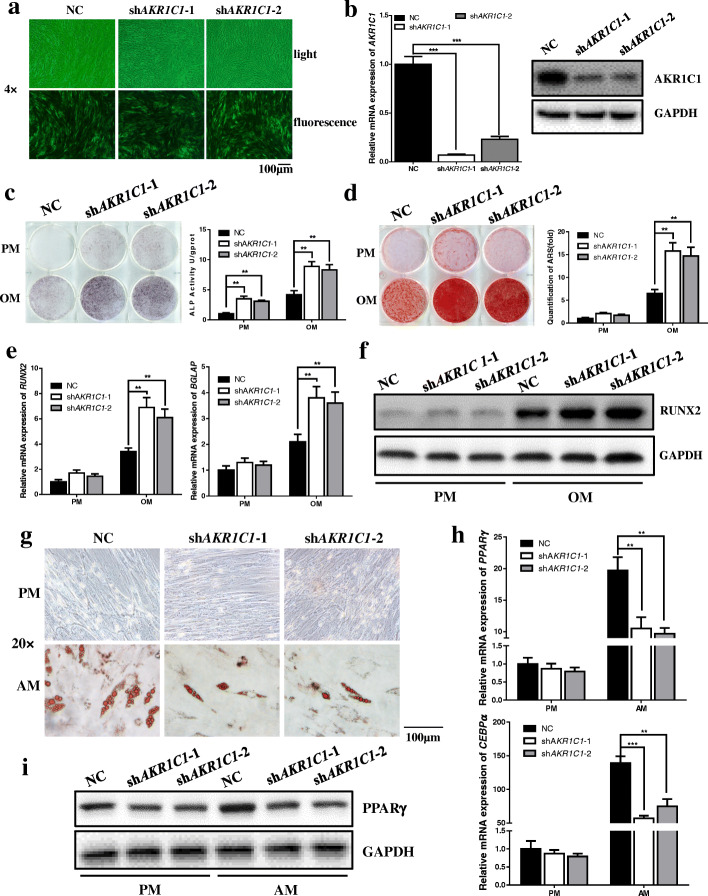
Knockdown of *AKR1C1* enhanced the osteogenic capacity and impaired the adipogenic capacity of hASCs in vitro. **a** Transfection efficiency showed by fluorescence microscopy. **b** Knockdown efficiency of AKR1C1 determined by qRT-PCR and western blot. **c** Knockdown of AKR1C1 enhanced the ALP activity after 7 days of osteogenic induction as shown by ALP staining and quantification. **d** Knockdown of AKR1C1 accelerated mineralization after 21 days of osteogenic induction as shown by ARS staining and quantification. **e** Knockdown of AKR1C1 promoted the mRNA expression of *RUNX2* on the 7th day of osteogenic induction and *BGLAP* on the 14th day of osteogenic induction as determined by qRT-PCR. **f** The protein expression of RUNX2 tested by western blot was consistent with the result of qRT-PCR. **g** Knockdown of AKR1C1 inhibited lipid droplet formation after 21 days of adipogenic induction as shown by oil red O staining. **h** Knockdown of AKR1C1 inhibited the mRNA expression of *PPARγ* and *CEBPα* on the 7th day of adipogenic induction as determined by qRT-PCR. **i** The protein expression of PPARγ tested by western blot was consistent with the result of qRT-PCR. **p < 0.01, ***p < 0.001

To reveal the role that AKR1C1 played in modulating of hASCs differentiation, we established stable *AKR1C1* knockdown hASCs. Two shRNA sequences targeting *AKR1C1* were used to avoid off-target effects. The transfection efficiency of lentivirus was confirmed by fluorescence microscopy (Fig. 2a). QRT-PCR and western blot (Fig. 2b) both showed that the expression of AKR1C1 was significantly knockdown by the two shRNA sequences. In addition, knockdown of AKR1C1 did not influence the proliferation of hASCs (Additional file: Figure S1). ALP staining and quantification revealed that AKR1C1 deficiency promoted the osteogenic differentiation of hASCs cultured in OM at day 7 (Fig. 2c), which was also observed in ARS staining and quantification (Fig. 2d). Besides, knockdown of AKR1C1 increased the mRNA expression of *RUNX2* at day 7 and *BGLAP* at day 14 (Fig. 2e) as well as the protein expression of RUNX2 at day 7 (Fig. 2f). On the other hand, deletion of AKR1C1 inhibited the adipogenic differentiation of hASCs cultured in AM at day 21 (Fig. 2g), as determined by oil red O staining. Meanwhile, knockdown of AKR1C1 decreased the mRNA expression of *PPARγ* and *CEBPα* at day 14 (Fig. 2 h) and western blot showed the consistent result (Fig. 2h).

Next, to further confirm the impact of AKR1C1 on differentiation of hASCs, we used plasmids to rescue the expression of AKR1C1 in AKR1C1 knockdown cells (sh*AKR1C1*-1 and sh*AKR1C1*-2). A previous study has proved that all the 3 AKR1C1 mutants, E127D, H222I, and R304L caused a significant loss of reductase activity [13]. As determined by CCK8 assay, re-expression of AKR1C1 had no significant effect on proliferation of hASCs both in sh*AKR1C1*-1 and sh*AKR1C1*-2 cells (Additional file: Figure S2).

**Fig. 3:**
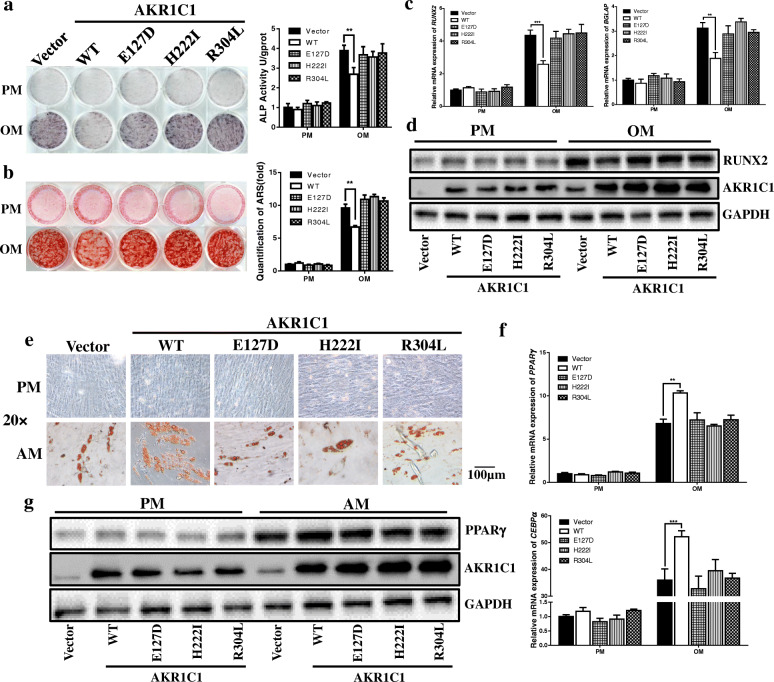
The regulation of AKR1C1 on hASCs differentiation in vitro depended on its enzyme activity. **a** ALP staining indicated that overexpression of AKR1C1 by wild type plasmid (WT) in the AKR1C1 knockdown cells (sh*AKR1C1*-1) downregulated the ALP activity whereas mutant plasmids (E127D, H222I and R304L) had no significant influence. The result of ALP quantification was consistent with the result of ALP staining. **b** The results of ARS staining and quantification were consistent with the results of ALP staining and quantification. **c** Overexpression of AKR1C1 by wild type plasmid (WT) in the AKR1C1 knockdown cells (sh*AKR1C1*-1) downregulated the mRNA expression of *RUNX2* and *BGLAP* whereas mutant plasmids (E127D, H222I and R304L) made no significant difference. **d** Western blot showed that transfection of wild type plasmid and mutant plasmids upregulated the protein expression of AKR1C1. The protein expression of RUNX2 was inhibited by transfection of wildtype plasmid but not mutant plasmids. **e** Oil red O staining revealed that in the AKR1C1 knockdown cells (sh*AKR1C1*-1), more lipid droplet formed in WT group, whereas mutant plasmids (E127D, H222I and R304L) made no significant difference. **f** Overexpression of AKR1C1 by wild type plasmid (WT) in the AKR1C1 knockdown cells (sh*AKR1C1*-1) upregulated the mRNA expression of *PPARγ* and *CEBPα* whereas mutant plasmids (E127D, H222I, and R304L) had no significant influence. **g** Western blot showed that transfection of wild type plasmid and mutant plasmids upregulated the protein expression of AKR1C1. The protein expression of PPARγ was promoted by transfection of wild type plasmid but not mutant plasmids. **p < 0.01, ***p < 0.001

ALP staining and quantification indicated that rescue of AKR1C1 by the wild type plasmid impaired the osteogenic capacity in AKR1C1 knockdown cells (sh*AKR1C1*-1), whereas transfection of 3 mutant plasmids which showed no enzyme activity of AKR1C1 did not have obvious effect (Fig. 3a). This phenomenon was also observed in ARS staining and quantification (Fig. 3b). The mRNA expression of *RUNX2* and *BGLAP* was also inhibited by transfection of wild type plasmid but not mutant ones (Fig. 3c). Besides, western blot (Fig. 3d) showed consistent results as determined by qRT-PCR. The similar results were obtained in sh*AKR1C1*-2 cells (Additional file: Figure S3).

As showed by oil red O staining, overexpression of AKR1C1 by the wild type plasmid rescued the adipogenic capacity in AKR1C1 knockdown cells (sh*AKR1C1*-1), whereas transfection of 3 AKR1C1 mutant plasmids did not have the same effect (Fig. 3e). In addition, the expression of *PPARγ* and *CEBPα* was also promoted by transfection of wild type plasmid but not mutant ones (Fig. 3f). Besides, western blot (Fig. 3g) showed that all kinds of plasmids significantly upregulated the expression of AKR1C1 and the expression of PPARγ was consistent with the result of qRT-PCR. The similar results were obtained in sh*AKR1C1*-2 cells (Additional file: Figure S4).

### AKR1C1 modulated the osteogenic and adipogenic differentiation of hASCs in vivo

**Fig. 4:**
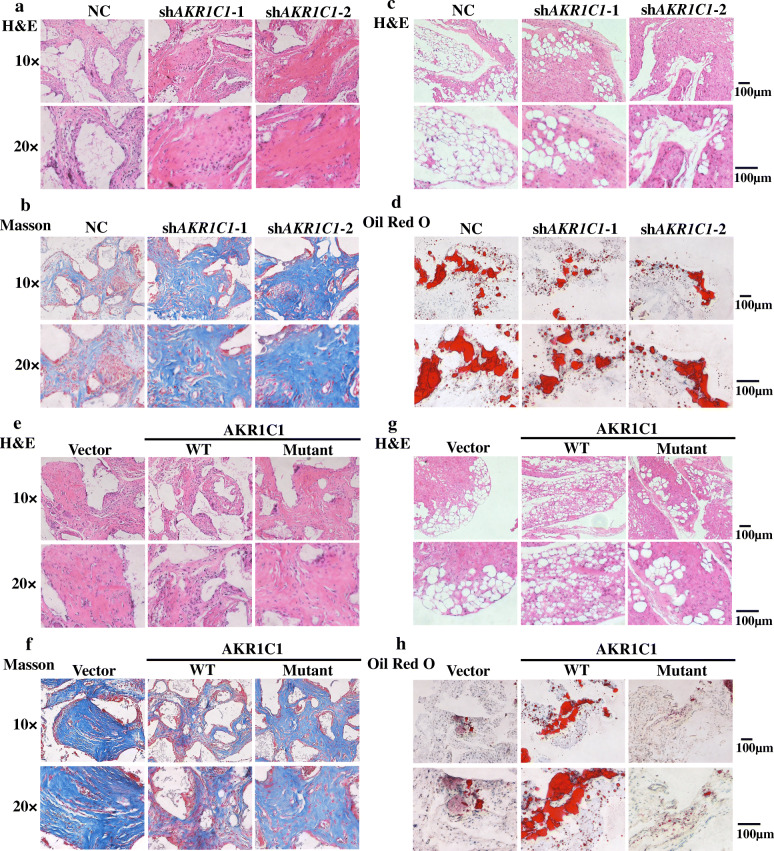
AKR1C1 regulated the osteogenic and adipogenic capacity of hASCs in vivo and the effect relied on the enzyme activity. **a** H&E staining of NC, sh*AKR1C1*-1, and sh*AKR1C1*-2 groups in heterotopic bone formation assay. **b** Masson staining of NC, sh*AKR1C1*-1, and sh*AKR1C1*-2 groups in heterotopic bone formation assay. **c** H&E staining of NC, sh*AKR1C1*-1, and sh*AKR1C1*-2 groups in heterotopic adipose tissue formation assay. Cells were separately loaded on Collagen Sponge scaffolds after 7 days of adipogenic induction and then implanted in the nude mice. **d** Oil red O staining of NC, sh*AKR1C1*-1, and sh*AKR1C1*-2 groups in heterotopic adipose tissue formation assay. **e** H&E staining of vector, wild type (WT) and mutant groups of AKR1C1 knockdown cells (sh*AKR1C1*-1) in heterotopic bone formation assay. **f** Masson staining of vector, wild type (WT) and mutant groups of AKR1C1 knockdown cells (sh*AKR1C1*-1) in heterotopic bone formation assay. **g** H&E staining of vector, wild type (WT) and mutant groups of AKR1C1 knockdown cells (sh*AKR1C1*-1) in heterotopic adipose tissue formation assay. Cells were separately loaded on Collagen Sponge scaffolds after 7 days of adipogenic induction and then implanted in the nude mice. **h** Oil red O staining of vector, wild type (WT), and mutant groups of AKR1C1 knockdown cells (sh*AKR1C1*-1) in heterotopic adipose tissue formation assay

As shown by the in vitro results, AKR1C1 exerted a vital regulatory effect on differentiation of hASCs. Thus, we tested whether AKR1C1 could influence the osteogenesis and adipogenesis capacity of hASCs in vivo. For heterotopic bone formation, three group of cells, NC, sh*AKR1C1*-1, and sh*AKR1C1*-2, were separately mixed with TCP carrier scaffolds and implanted in the nude mice (six mice per group). The samples were harvested 8 weeks after implantation. H&E staining showed that AKR1C1 knockdown cells formed more osteoid tissue (Fig. 4a, Additional file: Fig. S5a, c, d). As shown by Masson’s trichrome staining, there was more collagen organization (blue color) in the AKR1C1 knockdown groups compared to NC group (Fig. 4b, Additional file: Fig. S5b). For heterotopic adipose tissue formation, cells were separately loaded on Collagen Sponge scaffolds after 7 days of adipogenic induction and then implanted in the nude mice (six mice per group). Six weeks later, the samples were collected and analyzed. H&E (Fig. 4c) and oil red O (Fig. 4d, Additional file: Fig. S5i) staining both showed that fewer lipid droplets formed in AKR1C1 knockdown groups.

The in vitro experiments suggested that AKR1C1 regulated differentiation of hASCs via its enzyme activity, so we also examined whether AKR1C1 regulated differentiation of hASCs in vivo via its enzyme activity. Since that the three mutant plasmids all expressed AKR1C1 without enzyme activity successfully, E127D was chosen as the representative of the mutants in the following experiments. For heterotopic bone formation, sh*AKR1C1*-1 cells transfected with vector, wild type plasmid (WT) or E127D mutant plasmid were separately mixed with TCP carrier scaffolds and implanted in the nude mice (six mice per group). The samples were harvested 8 weeks after implantation. H&E staining showed that WT group formed less osteoid tissue than Vector group, whereas mutant plasmid made no significant difference (Fig. 4e, Additional file: Fig. S5e, g, h). As shown by Masson’s trichrome staining, there was less collagen organization (blue color) in the WT group than Vector group but no significant difference between Mutant and Vector group (Fig. 4f, Additional file: Fig. S5f). Similar phenomenon was observed in sh*AKR1C1*-2 cells (Additional file: Fig. S6). For heterotopic adipose tissue formation, sh*AKR1C1*-1 cells transfected with vector, wild type plasmid (WT) or E127D mutant plasmid were separately loaded on Collagen Sponge scaffolds after 7 days of adipogenic induction and then implanted in the nude mice (six mice per group). Six weeks later, the samples were collected and analyzed. H&E (Fig. 4g) and oil red O (Fig. 4h, Additional file: Fig. S5j) staining both showed that more lipid droplets formed in WT group but not Mutant group. Similar results were obtained in sh*AKR1C1*-2 cells (Additional file: Fig. S7).

### Knockdown of AKR1C1 promoted the osteogenic differentiation of hASCs in vitro through targeting PGR

**Fig. 5:**
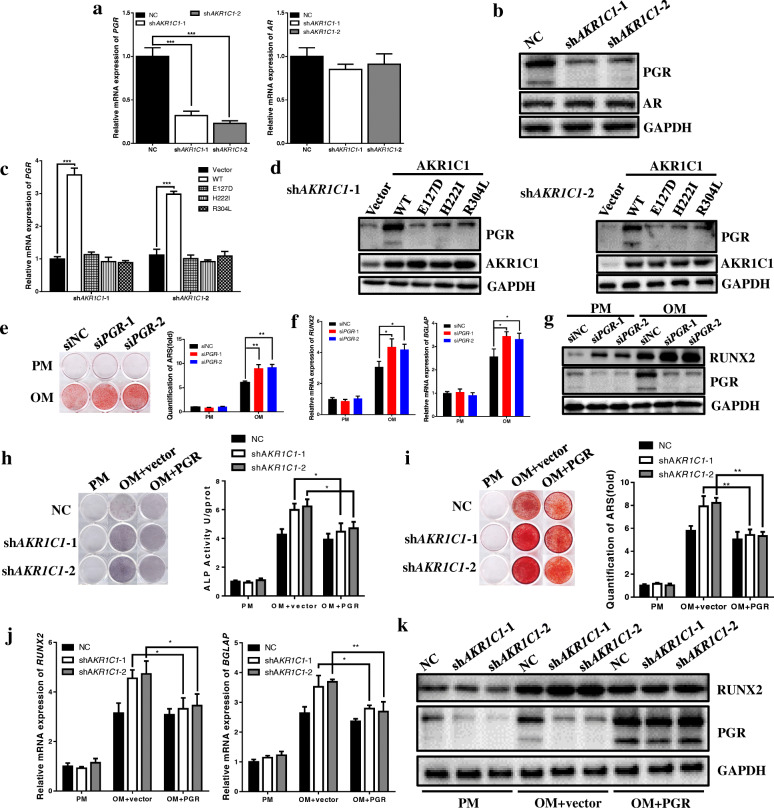
Knockdown of AKR1C1 promoted osteogenesis of hASCs in vitro through targeting PGR. **a**, **b** AKR1C1 knockdown inhibited expression of PGR but not AR validated by qRT-PCR and western blot. **c**, **d** QRT-PCR and western blot indicated that overexpression of AKR1C1 by wild type plasmid (WT) in the AKR1C1 knockdown cells upregulated expression of PGR whereas mutant plasmids (E127D, H222I, and R304L) had no significant influence. **e** ARS staining and quantification showed PGR knockdown in ASCs accelerated mineral accumulation after 21 days of osteogenic induction. **f**, **g** QRT-PCR and western blot showed that PGR knockdown in ASCs promoted expression of RUNX2 and BGLAP after 14 days of osteogenic induction. **h** Expression of PGR in *AKR1C1* knockdown cells slightly downregulated the ALP activity. **i** Expression of PGR in *AKR1C1* knockdown cells slowed down mineral accumulation. **j**, **k** QRT-PCR and western blot showed that expression of PGR in *AKR1C1* knockdown cells downregulated the expression of osteogenic markers (RUNX2, 7 day and BGLAP, 14 day). *p < 0.05, **p < 0.01, ***p < 0.001

Next, to explore the underlying mechanism how AKR1C1 modulate the differentiation of hASCs, NC and sh*AKR1C1* cells were subjected to western blot analysis for two key regulators closely related to both AKR1C1 and osteogenesis, PGR, and AR. Surprisingly, deletion of AKR1C1 significantly inhibited expression of PGR but not AR (Fig. 5a, b). Meanwhile, wild type plasmid of AKR1C1 rescued expression of PGR in AKR1C1 knockdown cells, but 3 mutant plasmids did not have obvious effect (Fig. 5c, d).

Since previous studies demonstrated that PGR displayed complicated function in osteogenic differentiation [24], we first knocked down PGR by siRNA in hASCs to confirm the role PGR played in osteogenesis. Expression of markers of osteogenesis tested by qRT-PCR and western blot showed that knockdown of PGR significantly promoted osteogenesis after 14 days of osteogenic induction (Fig. 5f, g). Meanwhile, knockdown of PGR obviously accelerated mineralization after 21 days of osteogenic induction, as determined by ARS staining and quantification (Fig. 5e). These results validated that deletion of PGR could promoted osteogenic differentiation of hASCs.

To further confirm the role PGR played in AKR1C1 regulating osteogenesis of hASCs, we expressed PGR in AKR1C1 knockdown cells by transfection of plasmid. Western blot exhibited that PGR was over-expressed successfully (Fig. 5k). ALP staining and quantification indicated that deletion of AKR1C1 could no longer significantly promoted ALP activity with expression of PGR (Fig. 5h). Meanwhile, knockdown of AKR1C1 could not accelerate mineralization with expression of PGR, as determined by ARS staining and quantification (Fig. 5i). The result of qRT-PCR also validated that deletion of AKR1C1 could not obviously promote osteogenesis when PGR was expressed at the same time (Fig. 5j), which was consistent with western blot result (Fig. 5k).

## Discussion

In this study, we revealed that AKR1C1 was involved in both the osteogenic and adipogenic differentiation of hMSCs. It could inhibit the osteogenesis and promote the adipogenesis of hASCs through its enzyme activity both in vitro and in vivo. These indicated that AKR1C1 could serve as a negative regulator of hASCs osteogenic differentiation in vitro and in vivo. Mechanistically, knockdown of AKR1C1 promoted osteogenesis of hASCs through targeting PGR. These new findings provided a new potential target for gene modification of seed cells in bone tissue engineering and treatment of diseases related with differential disorders of hASCs. The various molecular targets have different biological function in vivo besides regulating osteogenic differentiation of MSCs. Thus, they may be suitable for different patients who are in need of bone regeneration. Finding new potential targets will provide us more choice to get improved treatment effect for patients in need.

As representatives of MSCs, both hBMMSCs and hASCs are frequently used seed cells in bone tissue engineering. But compared to hBMMSCs, hASCs has more extensive sources and caused less trauma. So, hASCs were used in this research.

The in vitro experiment showed that the expression of AKR1C1 fluctuate greatly during both osteogenic and adipogenic induction process. These results demonstrated that AKR1C1 was closely related with lineage commitment of ASCs and might be deeply involved in regulating the differentiation of ASCs. In the osteogenesis process, AKR1C1 expression presented changes towards to reverse directions. It was first upregulated in the early stage (the initial 10 days) and subsequently decreased during the osteogenesis of ASCs. On the 14th day of osteogenesis, the expression of AKR1C1 was significantly down regulated compared with PM group. This may be caused by the cells’ self-protection mechanism to avoid over differentiation and keep homeostasis. Eventually, AKR1C1 expression was inhibited in the late stage of osteogenesis. This was consistent with the expression of AKR1C1 in BMMSCs of OVX mice, which also reflected the state after a long-term bone activity [33].

The lost-and-gain-of-function experiments revealed that AKR1C1 could serve as a negative regulator of osteogenic and a positive regulator of adipogenic differentiation of hASCs both in vitro and in vivo via its enzyme activity. AKR1C1 presented opposite regulatory effect on osteogenesis and adipogenesis. It is well known that adipogenic differentiation is usually inhibited when osteogenic differentiation is promoted. Therefore, the positive effect of AKR1C1 on the adipogenic differentiation of hASCs strengthened its negative effect on the osteogenic differentiation of hASCs. Taken together, this evidence indicated that AKR1C1 could play a critical role in mediating the lineage commitment of hASCs and thus could be a potential choice for molecular target of seed cell modification in bone tissue engineering. Previous studies mainly focused on the role of AKR1C1 in tumors [13–16, 34]. Its function in MSCs differentiation has been rarely documented. Moreover, little evidence of the modulating effect of other AKR1 family members on ASCs differentiation was available in the previous studies. Recently, Yajing J et al. mentioned that AKR1C1 could influence the survival of acute myeloid leukemia cells through the abnormal mesenchymal stromal cells in vitro [34]. But mesenchymal stromal cells of acute myeloid patients are in an abnormal state which have uncertain disorders. Thus, our study is still the first to reveal the important role of AKR1C1 in regulating ASCs lineage commitment.

Our study revealed that PGR mediated the regulation of AKR1C1 on osteogenic differentiation of hASCs. Although the role PGR played in bone has been studied [24–26], the effect of AKR1C1 on PGR has not been reported. We are the first to point out the regulation of AKR1C1 on PGR and AKR1C1 regulated osteogenic differentiation of hASCs via targeting PGR. Meanwhile, it should be noted that there was no progesterone in any kind of culture medium used in this research (PM, OM, or AM). Additionally, ASCs cannot synthesize progesterone themself [35, 36]. So the regulation of AKR1C1 on PGR is independent of progesterone. Although the mechanism of action by which AKR1C1 modulates osteogenesis of hASCs has not been completely elucidated, it is mediated, at least partly by PGR.

Nevertheless, there are still limitations in this study. We only evaluated the in vivo function of AKR1C1 by heterotopic bone and adipose tissue formation assay in nude mice. To further confirm the regulation effect and therapeutic value of AKR1C1, AKR1C1 knockout mouse model and more disease models in animal are required.

Overall, our results demonstrated that AKR1C1 could serve as a negative regulator of hASCs osteogenic differentiation through its enzyme activity in vitro and in vivo via targeting PGR, suggesting that AKR1C1 could be used as a novel molecular target in bone tissue engineering and might have therapeutic value in treatment of diseases related with differential disorders of hASCs.

## Conclusion

Collectively, the current study demonstrates that AKR1C1 participates in osteogenic differentiation as a novel negative regulator both in vitro and in vivo through targeting PGR. Our study figures out a new important function of AKR1C1 in addition to the field of tumor as well as suggests its application value in bone tissue engineering and diseases related with differential disorders of hASCs.

## Supplementary Information

**Additional file 1: Figure S1** Knockdown of AKR1C1 did not influence the proliferation of hMSCs. Knockdown of AKR1C1 caused no significant differences in the proliferative capacities of the cells compared with NC cells during day 1 (1) to day 7 (7), as shown by the growth curve of cells. **Figure S2** Overexpression of AKR1C1 by wildtype and mutant plasmids did not influence the proliferation of hMSCs. **a** In AKR1C1 knockdown cells (sh*AKR1C1*-1), transfection of wild type and mutant plasmids caused no significant differences in the proliferative capacities of the cells compared with cells transfected with vector during day 1 (1) to day 7 (7), as shown by the growth curve of cells. **b** In AKR1C1 knockdown cells (sh*AKR1C1*-2), transfection of wild type and mutant plasmids caused no significant differences in the proliferative capacities of the cells compared with cells transfected with vector during day 1 (1) to day 7 (7), as shown by the growth curve of cells. **Figure S3** The regulation of AKR1C1 on hMSCs osteogenic capacity in vitro depended on its enzyme activity. **a** ALP staining indicated that overexpression of AKR1C1 by wild type plasmid (WT) in the AKR1C1 knockdown cells (sh*AKR1C1*-2) downregulated the ALP activity whereas mutant plasmids (E127D, H222I and R304L) had no significant influence. The result of ALP quantification was consistent with the result of ALP staining. **b** The results of ARS staining and quantification were consistent with the results of ALP staining and quantification. **c** Overexpression of AKR1C1 by wild type plasmid (WT) in the AKR1C1 knockdown cells (sh*AKR1C1*-2) downregulated the mRNA expression of *RUNX2* and *BGLAP* whereas mutant plasmids (E127D, H222I and R304L) made no significant difference. **d** Western blot showed that transfection of wild type plasmid and mutant plasmids upregulated the protein expression of AKR1C1. The protein expression of RUNX2 was inhibited by transfection of wild type plasmid but not mutant plasmids. *p < 0.05, **p < 0.01, ***p < 0.001. **Figure S4** The regulation of AKR1C1 on hMSCs adipogenic capacity in vitro depended on its enzyme activity. **a** Oil red O staining revealed that in the AKR1C1 knockdown cells (sh*AKR1C1*-2), more lipid droplet formed in WT group, whereas mutant plasmids (E127D, H222I and R304L) made no significant difference. **b** Overexpression of AKR1C1 by wild type plasmid (WT) in the AKR1C1 knockdown cells (sh*AKR1C1*-2) upregulated the mRNA expression of *PPARγ* and *CEBPα* whereas mutant plasmids (E127D, H222I and R304L) had no significant influence. **c** Western blot showed that transfection of wild type plasmid and mutant plasmids upregulated the protein expression of AKR1C1. The protein expression of PPARγ was promoted by transfection of wild type plasmid but not mutant plasmids. *p < 0.05. **Figure S5** Histomorphometry analysis of the hMSCs-scaffold hybrids in heterotopic bone and adipose formation assay. **a, c, d, e, g, h** Histomorphometry analysis according to the H&E staining images of bone formation assay. **b, f** Histomorphometry analysis according to the masson’s staining images of bone formation assay. **i, j** Histomorphometry analysis according to the oil red O staining images of adipose formation assay. *p < 0.05, **p < 0.01, ***p < 0.001 compared with NC or Vector. **Figure S6** AKR1C1 regulated the osteogenic capacity of hMSCs in vivo through its enzyme activity. **a** H&E staining of vector, wild type (WT) and mutant groups of AKR1C1 knockdown cells (sh*AKR1C1*-2) in heterotopic bone formation assay. **b, e, f** Histomorphometry analysis according to the H&E staining images. **c** Masson staining of vector, wild type (WT) and mutant groups of AKR1C1 knockdown cells (sh*AKR1C1*-2) in heterotopic bone formation assay. **d** Histomorphometry analysis according to the masson’s staining images. *p < 0.05, **p < 0.01 compared with Vector. **Figure S7** AKR1C1 regulated the adipogenic capacity of hMSCs in vivo through its enzyme activity. **a** H&E staining of vector, wild type (WT) and mutant groups of AKR1C1 knockdown cells (sh*AKR1C1*-2) in heterotopic adipose tissue formation assay. **b** Oil red O staining of vector, wild type (WT) and mutant groups of AKR1C1 knockdown cells (sh*AKR1C1*-2) in heterotopic adipose tissue formation assay. **c** Histomorphometry analysis according to the oil red O staining images. **p < 0.01 compared with Vector.

## Data Availability

The authors confirm that all data underlying the findings are fully available.

## Abbreviations

AKR1C1: Aldo-keto reductase family 1 member C1
ALP: Alkaline phosphatase
AM: Adipogenic medium
AR: Androgen receptor
ARS: Alizarin Red S
ASCs: Adipose-derived mesenchymal stromal/stem cells
BGLAP: Bone gamma-carboxyglutamic acid-containing protein
BMMSCs: Bone marrow-derived mesenchymal stromal/stem cells
BV/TV: Trabecular bone volume/tissue volume
CEBPα: CCAAT enhancer binding protein alpha
GAPDH: Glyceraldehyde 3-phosphate dehydrogenase
H&E: Hematoxylin and eosin
Micro-CT: Micro computed tomography
MSCs: Mesenchymal stem cells
OM: Osteogenic medium
PGR: Progesterone receptor
PM: Proliferation medium
PPARγ: Peroxisome proliferator activated receptor gamma
qRT-PCR: Quantitative reverse transcription PCR
RUNX2: Runt-related transcription factor 2
Tb.N: Trabecular number
Tb.Sp: Trabecular spacing
Tb.Th: Trabecular thickness
