# Supplementary material for: Aldo-keto reductase family 1 member C1 regulates the osteogenic differentiation of human ASCs by targeting the progesterone receptor
Source: Stem Cell Res Ther. 2021 Jul 7;12:383. doi: 10.1186/s13287-021-02425-3 (PMC8261971; doi:10.1186/s13287-021-02425-3)
Supplement: Supplementary file 1 — Additional file 1: Figure S1 Knockdown of AKR1C1 did not influence the proliferation of hMSCs. Knockdown of AKR1C1 caused no significant differences in the proliferative capacities of the cells compared with NC cells during day 1 (1) to day 7 (7), as shown by the growth curve of cells. Figure S2 Overexpression of AKR1C1 by wildtype and mutant plasmids did not influence the proliferation of hMSCs. a In AKR1C1 knockdown cells (shAKR1C1-1), transfection of wild type and mutant plasmids caused no significant differences in the proliferative capacities of the cells compared with cells transfected with vector during day 1 (1) to day 7 (7), as shown by the growth curve of cells. b In AKR1C1 knockdown cells (shAKR1C1-2), transfection of wild type and mutant plasmids caused no significant differences in the proliferative capacities of the cells compared with cells transfected with vector during day 1 (1) to day 7 (7), as shown by the growth curve of cells. Figure S3 The regulation of AKR1C1 on hMSCs osteogenic capacity in vitro depended on its enzyme activity. a ALP staining indicated that overexpression of AKR1C1 by wild type plasmid (WT) in the AKR1C1 knockdown cells (shAKR1C1-2) downregulated the ALP activity whereas mutant plasmids (E127D, H222I and R304L) had no significant influence. The result of ALP quantification was consistent with the result of ALP staining. b The results of ARS staining and quantification were consistent with the results of ALP staining and quantification. c Overexpression of AKR1C1 by wild type plasmid (WT) in the AKR1C1 knockdown cells (shAKR1C1-2) downregulated the mRNA expression of RUNX2 and BGLAP whereas mutant plasmids (E127D, H222I and R304L) made no significant difference. d Western blot showed that transfection of wild type plasmid and mutant plasmids upregulated the protein expression of AKR1C1. The protein expression of RUNX2 was inhibited by transfection of wild type plasmid but not mutant plasmids. *p < 0.05, **p < 0.01, ***p < 0.00 [file 13287_2021_2425_MOESM1_ESM.docx]

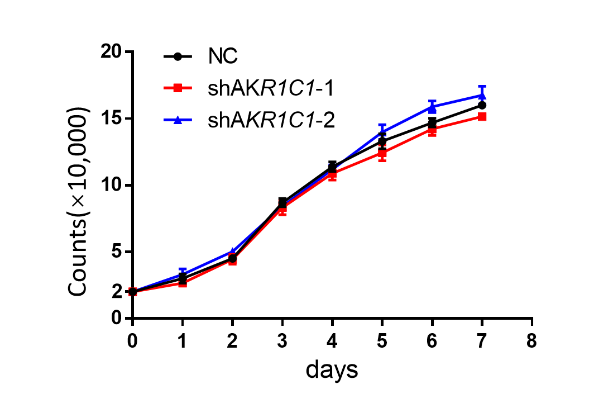


Figure S1 Knockdown of AKR1C1 didn’t influence the proliferation of hMSCs. Knockdown of AKR1C1 caused no significant differences in the proliferative capacities of the cells compared with NC cells during day 1 (1) to day 7 (7), as shown by the growth curve of cells.


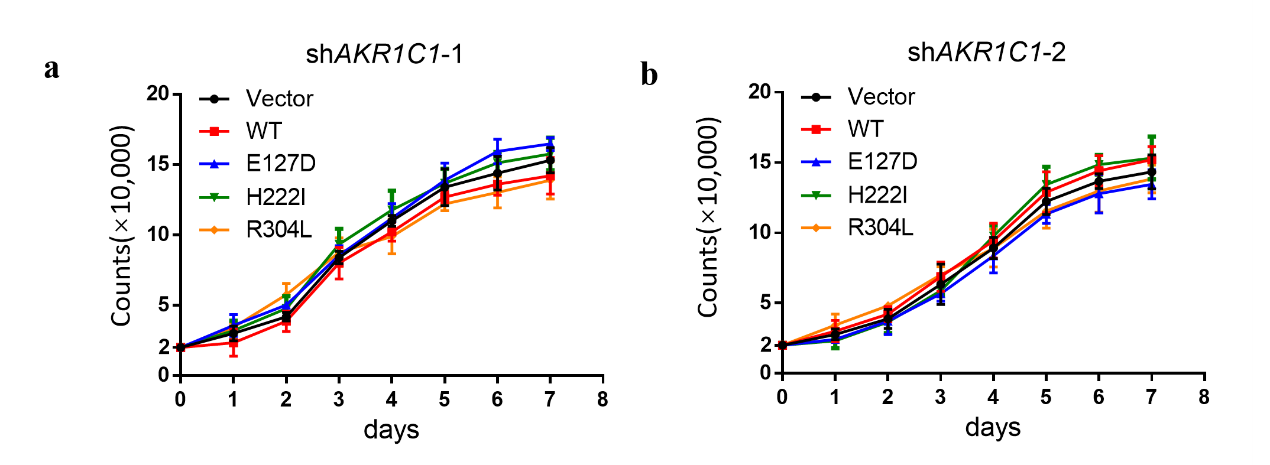


Figure S2 Overexpression of AKR1C1 by wild type and mutant plasmids didn’t influence the proliferation of hMSCs. **a** In AKR1C1 knockdown cells (sh*AKR1C1*-1), transfection of wild type and mutant plasmids caused no significant differences in the proliferative capacities of the cells compared with cells transfected with vector during day 1 (1) to day 7 (7), as shown by the growth curve of cells. **b** In AKR1C1 knockdown cells (sh*AKR1C1*-2), transfection of wild type and mutant plasmids caused no significant differences in the proliferative capacities of the cells compared with cells transfected with vector during day 1 (1) to day 7 (7), as shown by the growth curve of cells.


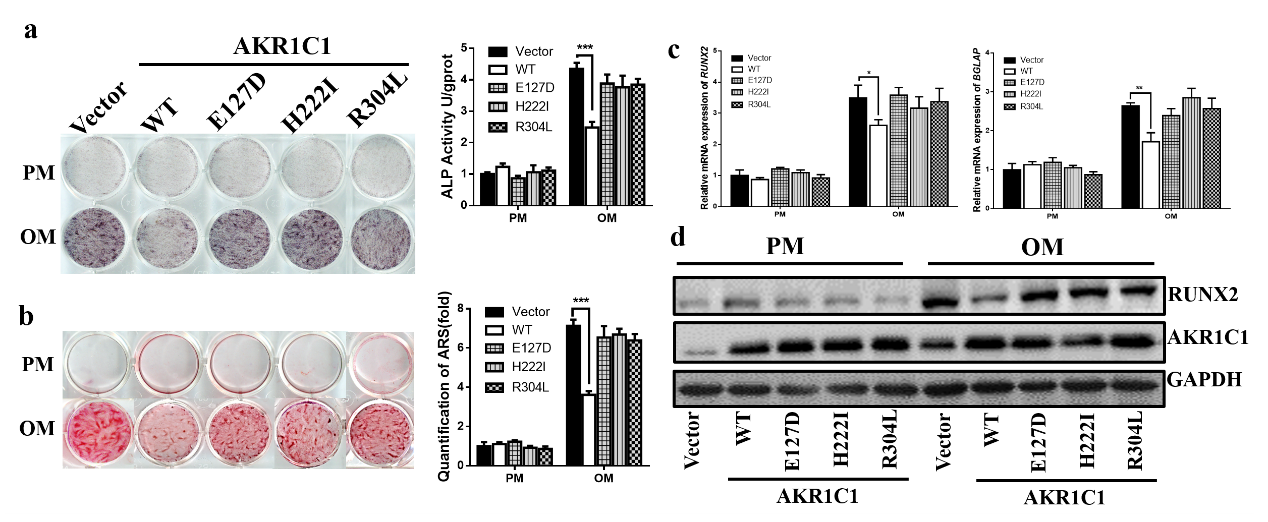


Figure S3 The regulation of AKR1C1 on hMSCs osteogenic capacity *in vitro* depended on its enzyme activity. **a** ALP staining indicated that overexpression of AKR1C1 by wild type plasmid (WT) in the AKR1C1 knockdown cells (sh*AKR1C1*-2) downregulated the ALP activity whereas mutant plasmids (E127D, H222I and R304L) had no significant influence. The result of ALP quantification was consistent with the result of ALP staining. **b** The results of ARS staining and quantification were consistent with the results of ALP staining and quantification. **c** Overexpression of AKR1C1 by wild type plasmid (WT) in the AKR1C1 knockdown cells (sh*AKR1C1*-2) downregulated the mRNA expression of *RUNX2* and *BGLAP* whereas mutant plasmids (E127D, H222I and R304L) made no significant difference. **d** Western blot showed that transfection of wild type plasmid and mutant plasmids upregulated the protein expression of AKR1C1. The protein expression of RUNX2 was inhibited by transfection of wild type plasmid but not mutant plasmids. **p*<0.05, ***p*<0.01, ****p*<0.001.


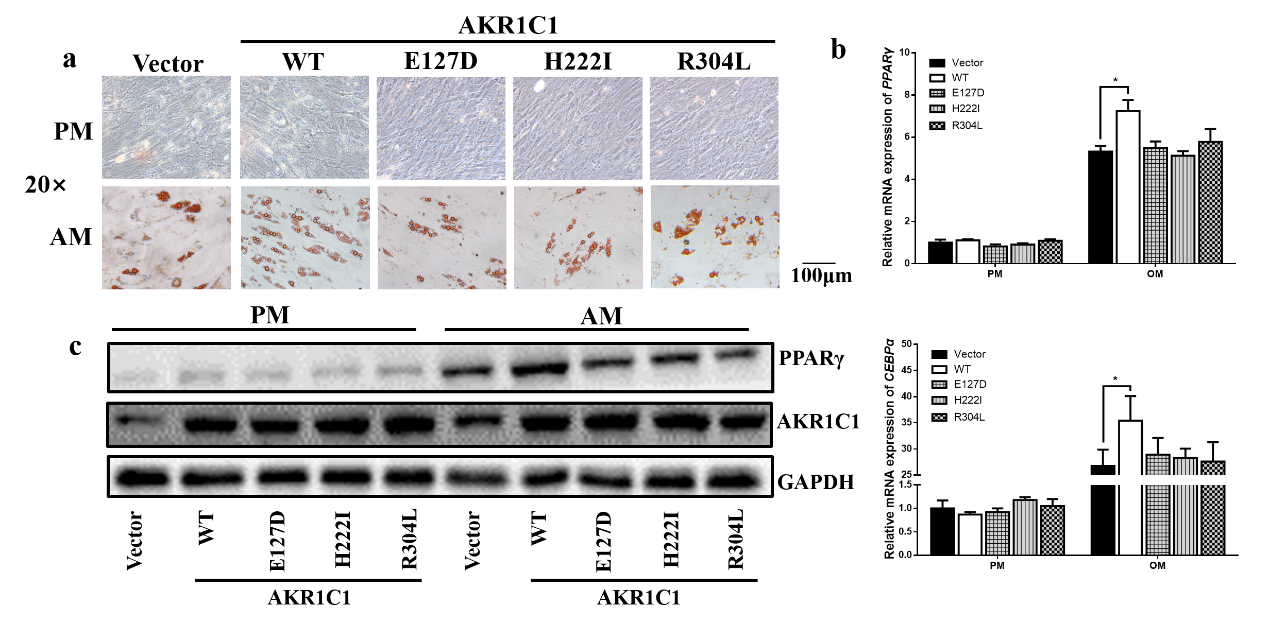


Figure S4 The regulation of AKR1C1 on hMSCs adipogenic capacity *in vitro* depended on its enzyme activity. **a** Oil red O staining revealed that in the AKR1C1 knockdown cells (sh*AKR1C1*-2), more lipid droplet formed in WT group, whereas mutant plasmids (E127D, H222I and R304L) made no significant difference. **b** Overexpression of AKR1C1 by wild type plasmid (WT) in the AKR1C1 knockdown cells (sh*AKR1C1*-2) upregulated the mRNA expression of *PPARγ* and *CEBPα* whereas mutant plasmids (E127D, H222I and R304L) had no significant influence. **c** Western blot showed that transfection of wild type plasmid and mutant plasmids upregulated the protein expression of AKR1C1. The protein expression of PPARγ was promoted by transfection of wild type plasmid but not mutant plasmids. **p*<0.05.


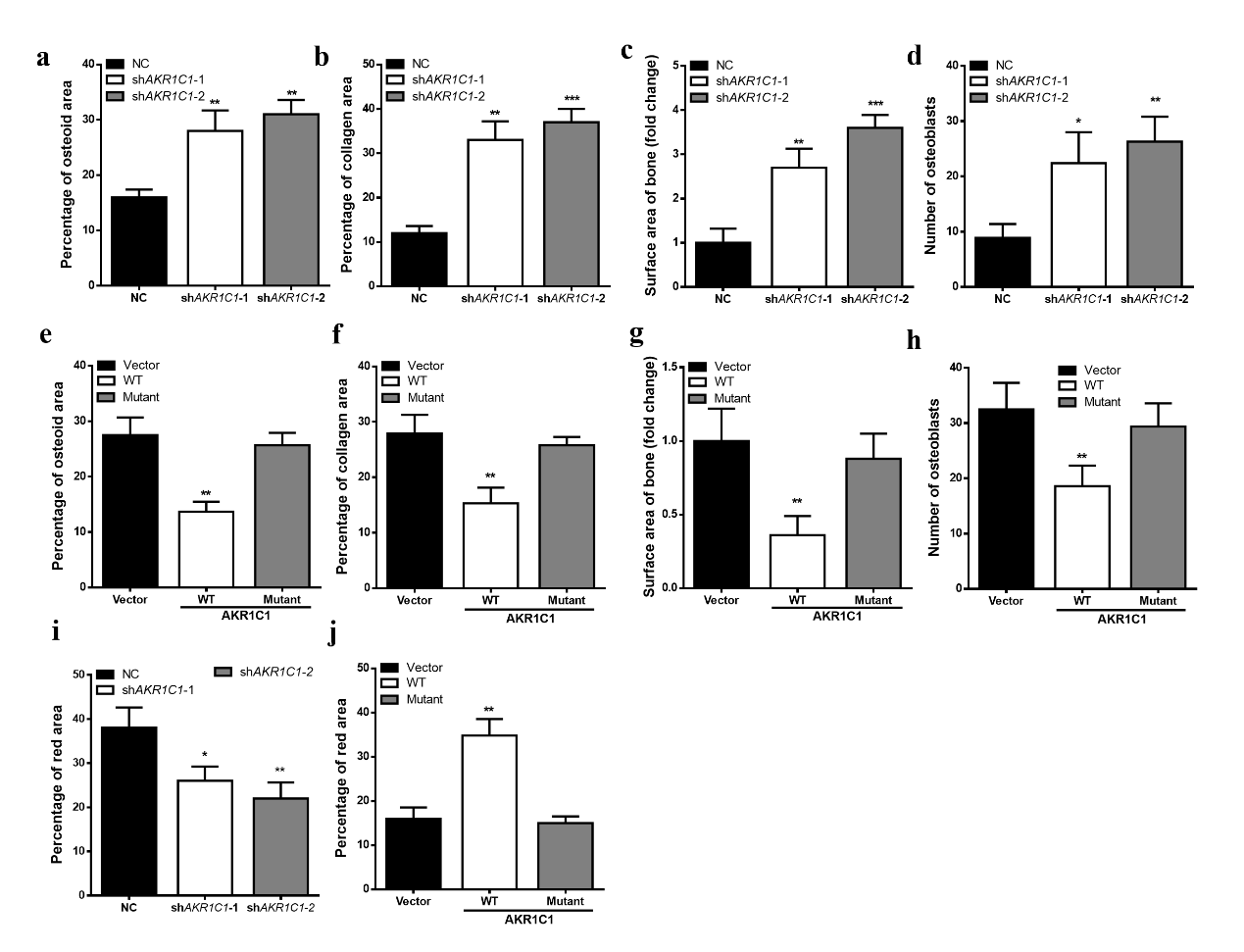


Figure S5 Histomorphometry analysis of the hMSCs-scaffold hybrids in heterotopic bone and adipose formation assay. **a, c, d, e, g, h** Histomorphometry analysis according to the H&E staining images of bone formation assay. **b, f** Histomorphometry analysis according to the masson’s staining images of bone formation assay. **i, j** Histomorphometry analysis according to the oil red O staining images of adipose formation assay. **p*<0.05, ***p*<0.01, ****p*<0.001 compared with NC or Vector.


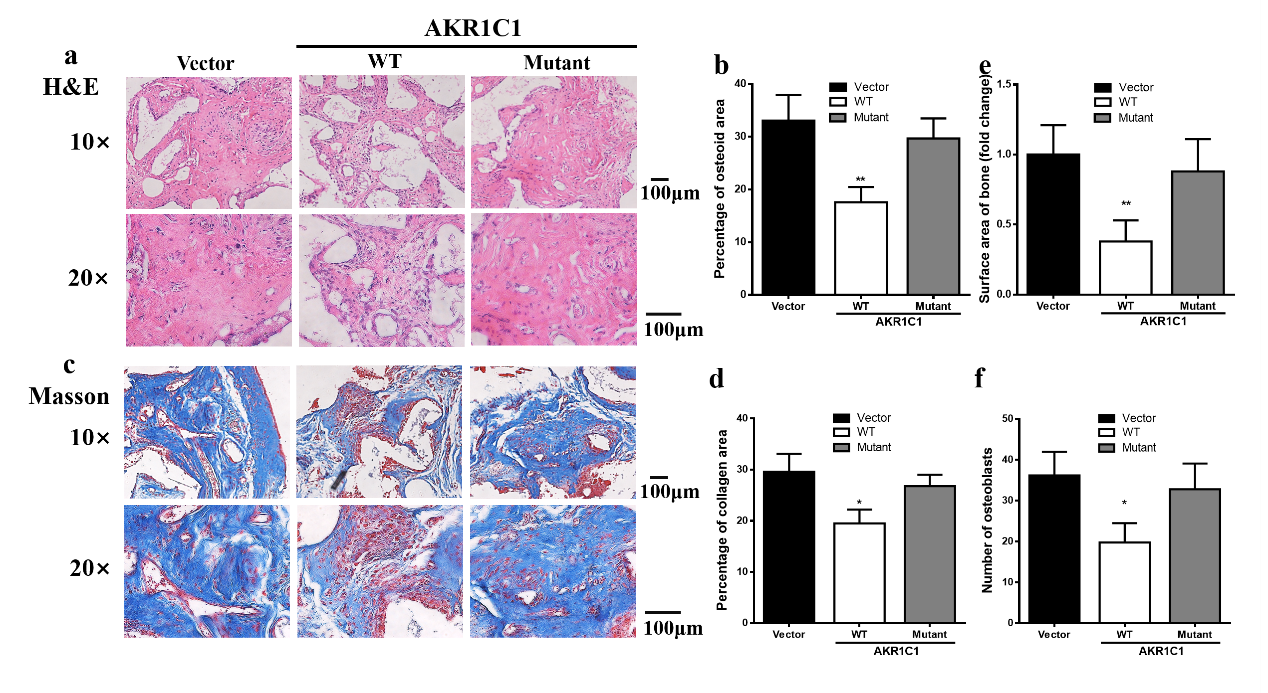


Figure S6 AKR1C1 regulated the osteogenic capacity of hMSCs *in vivo* through its enzyme activity. **a** H&E staining of vector, wild type (WT) and mutant groups of AKR1C1 knockdown cells (sh*AKR1C1*-2) in heterotopic bone formation assay. **b, e, f** Histomorphometry analysis according to the H&E staining images. **c** Masson staining of vector, wild type (WT) and mutant groups of AKR1C1 knockdown cells (sh*AKR1C1*-2) in heterotopic bone formation assay. **d** Histomorphometry analysis according to the masson’s staining images. **p*<0.05, ***p*<0.01 compared with Vector.


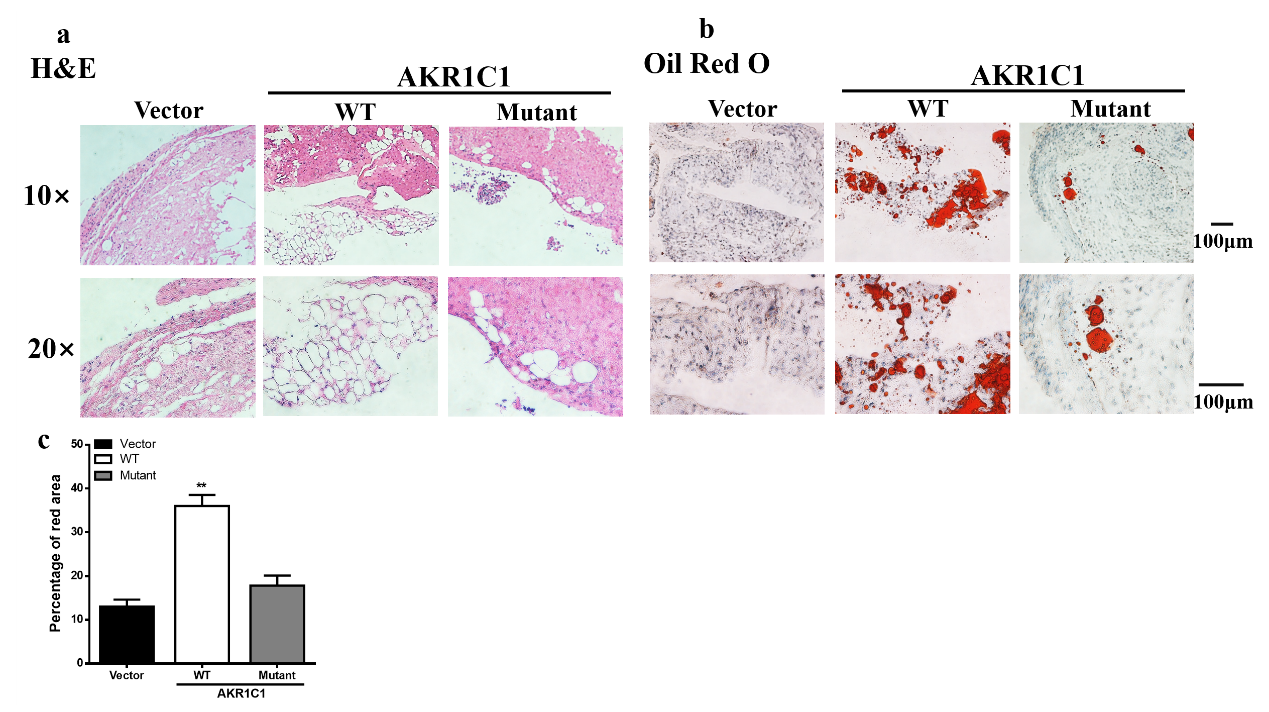


Figure S7 AKR1C1 regulated the adipogenic capacity of hMSCs *in vivo* through its enzyme activity. **a** H&E staining of vector, wild type (WT) and mutant groups of AKR1C1 knockdown cells (sh*AKR1C1*-2) in heterotopic adipose tissue formation assay. **b** Oil red O staining of vector, wild type (WT) and mutant groups of AKR1C1 knockdown cells (sh*AKR1C1*-2) in heterotopic adipose tissue formation assay. **c** Histomorphometry analysis according to the oil red O staining images. ***p*<0.01 compared with Vector.
